# Thirty-Sixth Annual Meeting—Mississippi Valley Association of Dental Surgeons

**Published:** 1880-03

**Authors:** E. G. Betty


### THIRTY-SIXTH ANNUAL MEETING—MISSISSIPPI VALLEY ASSOCIATION OF DENTAL SURGEONS.

The thirty-sixth yearly assembling of this body, took place in the Dental College lecture room on Tuesday, March 2nd, Pres't J. S. Cassidy, presiding; the meeting was called to order at 11 o'clock, A. M. by the President, and after prayer by Dr. Leslie, the minutes of last year's meeting were read by the Secretary, and no objections being offered, they stood approved as read. Reports from Committees being in order, Dr. J. Taft, Chairman of the Executive Committee, submitted the following programme of subjects for consideration by the society:

1st. Treatment and management of exposed tooth pulp.

2nd. Professional culture,—scientific and æsthetic.

3rd. Is it important that dental students should understand histological pathalogy?

4th. Is neuralgia a functional affection, or does it involve structural change of nerve tissue?

5th. Is the treatment of inflamed or sensitive dentine by escharotics in accordance with true physiology?

6th. Can thermal changes effect injuriously gold fillings?

7th. The progress of practical dentistry,–Operative,–Mechanical.

8th. With what therapeutic principles is the true dentist more especially interested? Give examples of agents pertaining to each, with their mode of action.

J. TAFT,<br>

J. TAYLOR,<br>

A. O. RAWLS, } *Executive Committee.*

On motion of Dr. E. G. Betty, the report as read, was accepted by the Association, with the usual reserved right to discuss any other subject that might be brought before the meeting. Dr. J. Taft's motion to place the hours of assembling at 9 to 12 M.; 2 to 5:30 P. M., and evening session as per appointment, respectively, was adopted by the Association, the afternoon of first day, excepted on account of meeting of the College Association.

In the absence of the Treasurer, Dr. J. G. Cameron, the Secretary was instructed to receive dues from members. The Committee on Membership proposed Drs. T. S. Brown of Gallipolis, O.; Otto Arnold, Ironton, O.; H. L. Moore, Batavia, O., and W. D. Kempton of Cincinnati, all of whom were elected by the Secretary's ballot. Dr. J. Taft's motion to appoint a committee of three, to draft resolutions indicative of the regret of this society, at the death of Dr. S. S. White, resulted in the appointment by the Chair of Drs. J. Leslie, J. Taft and G. W. Keely. No further business presenting itself for action by the Association, a motion to adjourn till 2 o'clock was carrted.

### AFTERNOON SESSION.

The College Association having postponed its meeting till the latter part of the afternoon, the Dental Association was then called to order by Pres't Cassidy, at 2:30 P. M. The minutes of morning's session were read and approved. The first subject on the programme, "Treatment and Management of Exposed Tooth-pulp," was announced for discussion, and Dr. J. Taft came to the front giving a comprehensive introduction of the topic, Drs. F. A. Hunter, H. A. Smith, G. W. Keely, A. O. Rawls, J. K. Jameson, Clayton and Reid, participating in the debate till the subject was passed. On motion the Association adjourned at 4 o'clock, to meet at 9 A. M., March 3rd.

### SECOND DAY.

### MORNING SESSION.

The meeting was called to order at 9:30 o'clock, Pres't Cassidy in the Chair, the minutes of afternoon session, first day, were read and on motion approved. The Treasurer, Dr. J. G. Cameron made his report, by which it was shown that $62.00 had been received as dues and initiation fees: $34.25 were expended for current expenses, leaving a balance of $28.75 in the treasury. On motion of Dr. J. Taft, the Chair appointed Drs. C. R. Taft and W. H. Sillito as auditing committee.

The second subject on the programme, "Professional Culture, scientific and æsthetic," having been announced, Dr. J. Taft opened the debate, Drs. Jas. Taylor, H. M. Reid, Berry, H. A. Smith and How, all participating in the talk. The Committee on Membership proposed Dr. J. S. McCampbell of Xenia, O., the gentleman being elected by the Secretary's ballot.

Adjourned to meet at 2 o'clock.

### AFTERNOON SESSION.

According to adjournment the society came to order, Vice Pres't Jay, presiding: minutes of morning session read and approved. The consideration of the second topic was resumed, Drs. J. Taft, Harlan, G. W. Keely, Sage and Leslie, making the concluding remarks. On motion, the subject was passed, as was also the third, making way for the fourth, "Is Neuralgia a functional affection, or does it involve structural change of Nerve Tissue?"

Dr. J. Taft's opening remarks were supplemented by those of Drs. Osmond, Rawls, J. Taylor and others, the subject was passed on motion and the fifth taken up. Dr. Jas. Taylor opened the discussion, as he put it on the programme to get the views of the different members, Drs. Osmond, Rawls, Berry and J. Taft expressing their opinions respectively.

Adjourned till 9 o'clock, A. M., March 4th.

### THIRD DAY.

### MORNING SESSION.

The Association assembled at 9:30 o'clock, Pres't Cassidy in the Chair, minutes of previous session were read and on motion accepted. The auditing committee reported the treasurer's accounts to be correct, which report was on motion, accepted and the committee discharged. The sixth topic was then announced for discussion by the Chair, Dr. J. Taft, as usual, opening the ball, Drs. J. Taylor, C. R. Taft, Rawls, Osmond, Porre, How, Berry, Jay and Arnold, all having something to say.

The Committee on Membership presented Dr. M. H. Fletcher for membership, he was unanimously elected. Dr. R. J. Porre, a delinquent member, was re-instated upon payment of two dollars. The seventh subject on the programme was then put before the house, Dr. J. W. Jay introducing the topic, after which it resolved into an informal discussion in the nature of incidents of office practice, Drs. Osmond, Kempton, Jay, Porre and J. Taft, each relating something or other. A motion was carried instructing the treasurer to pay the Janitor six dollars for services rendered the society. Adjourned to re-assemble at 2 o'clock.

### THIRD DAY.

### AFTERNOON SESSION.

Society called to order at 2:30 o'clock, Vice Pres't Jay in the Chair, minutes of previous session read, following which the consideration of the seventh subject was resumed by Drs. J. Taft, Osmond, Van Antwerp, G. W. Keely, Cameron, Rehwinkel, Hunter, Ulery and others. On motion, this topic was passed and the eighth laid over.

The committee appointed to draft resolutions on the death of Dr. S. S. White, reported the following:

"Your committee appointed to take into consideration the decease of Dr. Samuel S. White, respectfully submit. That—While death is common, and we are often called to mourn; yet when an eminent man and loved friend is suddenly removed, we realize how difficult it is to express fully the sense of our loss. When the sad tidings of the death of our dear and honored friend Dr Samuel S. White reached us, we felt that a great man had been called to a higher sphere. We knew he had gone abroad to rest awhile from the weariness and weakness induced by labor, largely rendered in behalf of the Dental Profession.

We rembember the days and nights during his earlier years that he spent in his humble laboratory, mixing and compounding minerals and wresting from the oxides of metals, the secrets of their colors in new relations, determined to bring forth from his muffle an artificial tooth more like the natural tooth, than yet had been produced by the genius of man. We remember the bright young man and how his manly face lit up with the glow of genius at the successive improvements his repeated experiments demonstrated. What a poor and mistaken estimate we would have of him, if we should forget this love of the beautiful and exact in nature, that animated him to struggle with material things for all her delicate tints in the thousands of his experiments.

The results of his laborious devotion to produce that great want of his day in the dental profession, an improved artificial tooth, were crowned with success. He might have continued his dental practice and doubtless would have attained eminence as a practitioner, but notwithstanding experiments were costly and returns small, he persevered and secured for the usefulness and beauty of our race —a ceramic tooth, that has enabled skillful men to demonstrate that his and our noble profession is at once a beautiful science and wonderful art. His knowledge of dental needs in every department suggested additional improvements, and by his own improvements and his readiness to encourage others, he largely assisted in bringing before the profession many things that he deemed of use. At every gathering of the world's industries that have been held for almost thirty years in the various nations of the earth, the juries of each in his special department, have awarded to him the highest awards, thereby confirming the American verdict that he is of all our worthy and working compeers, the man whom we most delight to honor, whose untimely death we most profoundly regret. His zeal and interest in dental education are well known.

This Association being almost the oldest known, was often made the medium of his discoveries and improvements, and the OHIO DENTAL COLLEGE enlisted his liberality, his assistance in taking several shares of stock was rendered at a time, when the struggles of its youth needed help, and we know that in his death, it, like other dental colleges, has lost a learned, liberal and judicious friend. His generosity was as proverbial as his honesty; the truthfulness that beamed from his eye and was even on his tongue, was as pleasant as his presence was always gentle, manly, considerate and kind. Such was largely the earthly side of our departed friend, and many of us who know of his thoughts and communings that rise above the common links of trade, realized that the divine side that manifested itself in honesty to his God, his love for man, produced a combination of excellences of character most worthy of our imitation. To his dear family we offer this honest tribute in testimony of his sterling worth and deep regret for the death of the beloved husband and dear father, and during their hours of poignant grief, we affectionately tender them our deep sympathy in this—their painful bereavement.

JAS. LESLIE,<br>

J. TAFT,<br>

G. W. KEELY, } *Committee.*

Resolved, that a copy of the above be sent to the family and published in the dental journals.

The report from the committee was adopted by the Association on a unanimous rising vote.

Bills amounting to $7.00 for printing and postage were ordered paid. An appropriation of $15.00 was made for Secretary's services.

The election of officers being next in order, the Association proceeded to ballot for candidates with the following result:

Pres't, H. M. Reid, Cincinnati, O.; 1st Vice Pres't, Wm. Van Antwerp, Mt. Sterling, Ky.; 2nd Vice Pres't, N. S. Hoff, Cincinnati, O.; Recording Sec'y, E. G. Betty, Cincinnati, O.; Corresponding Sec'y, C. I. Kelly, Oxford, O.; Treasurer, J. G. Cameron, Cincinnati, O.

The Pres't elect was installed in his office, and the retiring Pres't, J. S. Cassidy, made a short but interesting address which was received with applause. On motion of Dr. W. S. How, a vote of thanks was tendered the retiring executive for his efficient discharge of the duties of his office. On motion of Jas. Taylor, Dr. Cassidy's address was referred to the publication committee for publication in the Dental Register. On motion, Drs. J. Taft and N. S. Hoff, were elected a committee with power to appoint delegates to the American Dental Association. A motion was carried empowering Drs. G. W. Keely, J. Taft and F. H. Rehwinkel, as a committee to draft suitable resolutions relative to the death of Dr. W. H. Shadoan of Louisville, Ky. The committee's report as follows, was unanimously adopted.

WHEREAS: This Association has learned with profound regret of the death, after a long and painful illness of W. H. Shadoan, D. D. S., graduate of the Ohio College of Dental Surgery.

THEREFORE, RESOLVED: That in the death of Dr. Shadoan, this society has lost an earnest and enthusiastic friend and co-laborer, one who, by his pen, used his best endeavors to advance and maintain the honor and advancement of Dental Science.

RESOLVED: That we accord to his memory this heartfelt tribute of respect, and tender to his surviving widow our condolence and sympathy in her great bereavement.

RESOLVED: That a copy of these resolutions be furnished the widow, and also published in the Dental Register.

G. W. KEELY,
J. TAFT,
F. H. REHWINKEL.

The Pres't, Dr. Reid, announced the following standing committees.

N. S. HOFF,

C. I. KEELY,

OTTO ARNOLD, } *Membership.*

R. J. PORRE,
T. S. BROWN,
H. L. MOORE, } *Ethics.*

F. H. REHWINKEL,

JAS. TAYLOR,

A. F. EMMINGER, } *Publication.*

G. W. KEELY,
C. R. TAFT, } *Executive.*
H. A. SMITH,

The Association then adjourned to meet again at 10 o'clock, A. M., on the first Wednesday in March, 1881.

E. G. BETTY, Rec. Sec'y.

